# Data Analytics for Smart Parking Applications

**DOI:** 10.3390/s16101575

**Published:** 2016-09-23

**Authors:** Nicola Piovesan, Leo Turi, Enrico Toigo, Borja Martinez, Michele Rossi

**Affiliations:** 1 Centre Tecnològic de Telecomunicacions de Catalunya (CTTC), Parc Mediterrani de la Tecnologia, Av. Carl Friedrich Gauss, 7, Castelldefels, 08860 Barcelona, Spain; nicola.piovesan@cttc.es; 2 Department of Information Engineering (DEI), University of Padova, Via Gradenigo 6/B, 35131 Padova, Italy; turileo.1218@gmail.com (L.T.); toigoenrico.91@gmail.com (E.T.); 3 Internet Interdisciplinary Institute (IN3), Universitat Oberta de Catalunya (UOC), Parc Mediterrani de la Tecnologia, Av. Carl Friedrich Gauss 5, Castelldefels, 08860 Barcelona, Spain; bmartinezh@uoc.edu

**Keywords:** data analytics, smart parking data, wireless sensing, Self-Organizing Maps (SOM), data clustering, Internet of Things

## Abstract

We consider real-life smart parking systems where parking lot occupancy data are collected from field sensor devices and sent to backend servers for further processing and usage for applications. Our objective is to make these data useful to end users, such as parking managers, and, ultimately, to citizens. To this end, we concoct and validate an automated classification algorithm having two objectives: (1) outlier detection: to detect sensors with anomalous behavioral patterns, i.e., outliers; and (2) clustering: to group the parking sensors exhibiting similar patterns into distinct clusters. We first analyze the statistics of real parking data, obtaining suitable simulation models for parking traces. We then consider a simple classification algorithm based on the empirical complementary distribution function of occupancy times and show its limitations. Hence, we design a more sophisticated algorithm exploiting unsupervised learning techniques (self-organizing maps). These are tuned following a supervised approach using our trace generator and are compared against other clustering schemes, namely expectation maximization, *k*-means clustering and DBSCAN, considering six months of data from a real sensor deployment. Our approach is found to be superior in terms of classification accuracy, while also being capable of identifying all of the outliers in the dataset.

## 1. Introduction

Large-scale Internet of Things (IoT) deployments are being massively installed within smart cities [1], and alongside their adoption, there is a concurrent need for advanced processing functionalities to handle the vast amount of data generated by sensor devices and, more importantly, to make these data useful for public administrations and citizens. IoT technology allows monitoring a wide range of physical objects through low-cost and possibly low-power sensing and transmission technologies. Nevertheless, despite the ever-growing interest for IoT, to date, there have been few technical investigations employing data analytics to solve real-world problems in smart cities and especially utilizing IoT data as a basis for new applications [2,3]. A detailed literature review on smart parking applications, technologies and algorithms is provided in the next Section 2.

Our focus in this paper is on data analysis tools for smart parking systems, and for our designs, tests and considerations, we use data from a large commercial smart parking deployment installed and maintained by Worldsensing (http://www.worldsensing.com/) in a town in Northern Italy. This deployment features 370 wireless sensor nodes that are placed underneath parking spaces to provide real-time parking availability measures. Readings from this Wireless Sensor Network (WSN) were collected over a period of six full months and used for the results that we present here.

Specifically, we design processing tools to extract relevant statistical features from real-life parking data with the ultimate goal of classifying parking spaces according to their spatio-temporal patterns. Besides this, we also provide a means to automatically detect outliers, i.e., to identify those sensors whose observations do not conform to expected patterns. These outliers may for example be malfunctioning nodes, which need to be detected for inspection and maintenance, or may pinpoint anomalous parking behaviors. Note that this classification is a key feature for parking managers and also reveals interesting aspects on how people move and their habits. For example, it is possible to label neighborhoods as residential or commercial by just looking at how parking spaces are used. This knowledge may indicate preferred locations for shops or other services or may be used to infer those routes that are likely to become congested due to people commuting. Therefore, besides managing parking spaces and detecting misconduct, further smart city applications can be built on top of our classification algorithms, by fusing what we learn with other types of data.

We start our work by discussing some statistical aspects of the Worldsensing dataset. As a first step toward the classification of parking data, we use empirically-derived distributions as a means to identify anomalous readings (outliers), adopting a naive approach. This method is however soon discarded, as it is incapable of jointly providing good classification accuracies (i.e., parking events are correctly labeled) and high detection rates (i.e., all of the events of a certain class are detected). Upon conducting this preliminary experiment, it quickly became apparent that a more sophisticated approach is required and also that parking data are rather complex as: (1) they feature different statistics across different days of the week and hours of each day and (2) multiple metrics are to be jointly tracked for a meaningful classification of parking spaces, such as the parking event duration, the vacancy duration and the frequency of parking events. As a next step, we thus jointly summarize these performance indicators into suitable feature vectors, which are then utilized in conjunction with selected clustering algorithms to classify parking signals.

As for the clustering schemes, the following algorithms from the literature are considered: (1) *k*-means; see, e.g., [4]; (2) Density-Based Spatial Clustering of Applications with Noise (DBSCAN) [5], which is the de facto standard unsupervised clustering algorithm; and (3) a clustering scheme based on Expectation Maximization (EM) [6], which is taken from a recent paper on clustering for smart parking data [7] and is here adapted to our specific settings. We additionally design an original clustering technique, by utilizing Self-Organizing Maps (SOM) [8,9], which are unsupervised neural networks possessing the ability of learning prototypes in multi-dimensional vector spaces. This is closely related to finding regions, one per prototype, and solving a multi-dimensional classification problem [10]. Our SOM-based clustering approach is presented in two flavors: (i) an SOM-based scheme requiring the number of data classes (clusters) to be known beforehand and (ii) an unsupervised SOM-based algorithm, that automatically finds the number of classes from the analysis of feature vectors.

All of the clustering algorithms are fine tuned following a supervised approach, using synthetic traces that closely resemble real signals. In this way, we obtain a ground truth dataset that is utilized to verify the correctness of the classifiers. Finally, six months of data from the Worldsensing deployment are used to comparatively assess the performance of all approaches. Overall, we found that *k*-means, DBSCAN and EM-clustering all show classification problems and fail to separate outliers from the other clusters, whereas our SOM-based scheme performs satisfactorily, reaching the highest classification performance when tested on synthetically-generated parking events and reliably detecting all outliers when applied to real data.

The remainder of this paper is organized as follows. The related work is discussed in Section 2. The system model, along with some preliminary analysis of the parking data, is presented in Section 3. The naive approach for the classification of parking spaces is explored in Section 4. The computation of features from parking data, along with the discussion of standard schemes and the presentation of our new SOM-based clustering algorithm are provided in Section 5. The considered approaches are fine tuned and numerically evaluated in Section 6, through synthetic and real datasets. Our final considerations are presented in Section 7.

## 2. Related Work

The usage of automated instrumentation for on-street parking monitoring [11] has become popular in several cities around the world. In existing deployments, small sensing devices are usually placed in every parking spot to monitor large urban areas. Representative examples are Los Angeles [12], San Francisco [13] and Barcelona [14], among many others. The first layer of these complex systems is comprised of on-street parking sensors, which are small-scale wireless devices used to monitor the presence of vehicles. Each sensor periodically wakes up to check the occupancy state of the assigned parking lot. As a car parks above it, the sensor detects its presence, and the event is wirelessly reported to a gateway within radio coverage. From the gateway, the data are then sent to backend servers for further processing, remote parking management and visualization.

The main goal of these systems is to improve the operation efficiency of public parking, which is achieved through the collection of fine-grained, constant and accurate information on parking lot occupancy. With this objective in mind, the collected data are analyzed and provided to the city parking management division through suitable dashboards. On top of this, the availability of real-time parking information also enables new services, providing an improved urban user experience. As an example, Parking Guidance and Information (PGI) systems [15] help drivers find parking spaces more efficiently, thus ameliorating the problem of cruising for curb parking [16]. On-street parking reservation is another relevant application example [17,18].

We underline that, despite the fast growing number of real-world installations, only a few scientific papers have appeared so far on data mining and information processing for smart parking. The use of big data analytics is discussed in [19] and urban social sensing in [20], where open challenges related to the required technology to collect, store, analyze and visualize large amounts of parking data are also investigated. An architectural framework for data collection and analysis is discussed in [21]. The authors of [2] discuss some parking problems in India, elaborating on the pros and cons of parking technology, as well as on its public acceptance.

The authors of [22] put forward a swarm intelligence-based vehicle parking system employing context awareness and wireless communications. In this system, parking areas are instrumented with web-based tools and with a wireless sensor infrastructure. Parking information is collected and visualized through suitable Internet-based dashboards and is communicated to the vehicles searching for parking spaces. The route to the nearest available parking lots is computed through particle swarm optimization algorithms and is sent to the drivers. Data mining for vehicular maintenance and insurance data are investigated in [23], experimenting with Bayes and logistic regression models. A recent paper [24] employs data from on-street parking sensors from the city of Santander, in Spain, to design and validate a framework for parking availability prediction (either by area or assessing the future state of specific parking spaces). Statistical properties of parking signals are explored in [25], where the authors propose the concept of lean sensing. Specifically, they trade some sensing accuracy at the benefit of improved operational costs: temporal and spatial correlations are then utilized to infer the system-state from incomplete parking availability information in an attempt at reducing the power consumption associated with sensing and reporting.

The work in [7] is, to the best of our knowledge, the only one that has appeared on data analytics for smart parking spaces. There, the authors investigate farthest first and EM clustering schemes to subdivide parking traces into clusters based on their statistical features and then use Support Vector Data Description (SVDD) to identify extreme behaviors (two classes), i.e., either abnormally high or abnormally low occupancy locations. In this paper, we consider the most advanced EM approach from [7], where the number of cluster is self-assessed based on the input data. We then compare this technique with other clustering schemes and with an original SOM-based algorithm that we devise.

## 3. System Model

In the following sections, we discuss the dataset that was used for the results, detailing some general properties of parking data and using a simple statistical model as the basis of an event-based simulator. This simulator is utilized for the design of the algorithms of Section 5 and their fine tuning.

### 3.1. Statistical Models for Parking Data

**Figure 1:**
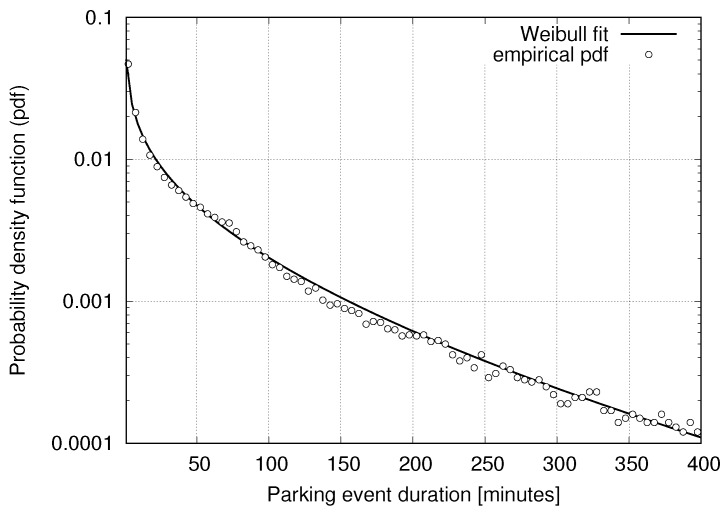
Probability distribution function of the parking duration (random variable (r.v.) $t_{\mathrm{ON}}$).

For the results in this paper, we use measurements from a WSN parking sensor deployment installed and managed by Worldsensing. The deployment is comprised of N=370 wireless sensors, where a node is located underneath each parking spot. Data were collected for six full months, from 1 December 2014 to 30 May 2015, counting more than one million parking events. With $s_{i}\left(t\right)\in \left\{0,1\right\}$, we denote the occupancy status of sensor *i* at the generic time *t*, where $s_{i}\left(t\right)=0$ and $s_{i}\left(t\right)=1$ respectively mean that the corresponding parking spot is vacant and busy at time *t*. In this paper, we are concerned with the statistics of parking events, namely their duration, which is modeled for sensor *i* through the non-negative random variable (r.v.) $t_{\mathrm{ON}}^{i}$, and the duration of vacancies, modeled through the non-negative r.v. $t_{\mathrm{OFF}}^{i}$. In what follows, for any parking space, we respectively refer to “ON” and “OFF” as the parking states corresponding to busy and vacant. With $T_{\mathrm{ON}}^{i}$ and $T_{\mathrm{OFF}}^{i}$, we indicate $T_{\mathrm{OFF}}^{i}=E\left[t_{\mathrm{OFF}}^{i}\right]$ and $T_{\mathrm{ON}}^{i}=E\left[t_{\mathrm{ON}}^{i}\right]$, respectively. In Figure 1, the empirical probability density function (pdf) of $t_{\mathrm{ON}}$ is plotted against a heavy-tailed Weibull distribution (similar results hold for $t_{\mathrm{OFF}}$). Although the empirical pdf shows an oscillatory pattern, which smooths out for increasing values of the abscissa, the Weibull nicely captures the general trend. These results are also confirmed by experimental data from the Smart Santander WSN deployment; see [24,26].

For a non-negative r.v. *Z*, the Weibull pdf is defined as:

(1) $$p_{Z}\left(z\right)=\frac{\kappa }{\lambda }{\left(\frac{z}{\lambda }\right)}^{\kappa -1}e^{-{\left(z/\lambda \right)}^{\kappa }}\;,\;z\geq 0\;,$$

where κ>0 is the shape parameter and λ>0 is the scale parameter. In Table 1, we provide these parameters for r.v.’s $t_{\mathrm{ON}}$ (parking duration) and $t_{\mathrm{OFF}}$ (duration of vacancies). The Weibull pdf of Figure 1 was obtained considering all possible parking events and is referred to here as “average”. In the table, we also detail the pdfs of the parking sensors with the longest (“Max”) and shortest (“Min”) events.

### 3.2. Event-Based Simulator

**Figure 2:**
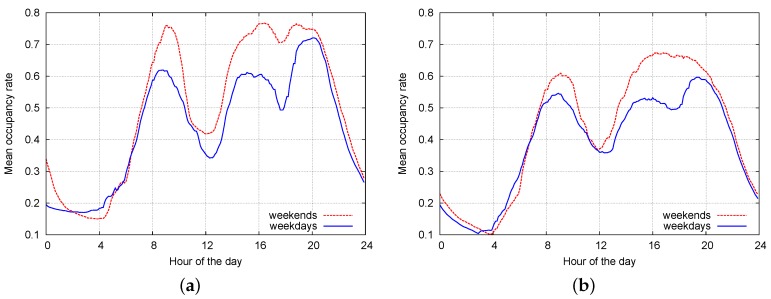
Holiday/weekend vs. weekday occupancy rates in the month of January 2015: comparison between occupancy rates from real data and synthetic traces generated by our event-based simulator. (**a**) Worldsensing dataset; (**b**) event-based simulator.

An event-based simulator was built with the aim of designing the classification algorithms and performing their fine tuning. For each sensor in the Worldsensing deployment, we obtained the corresponding empirical pdfs for $t_{\mathrm{ON}}$ and $t_{\mathrm{OFF}}$, for each hour of the day (48 pdfs) and for each day of the week. Thus, parking durations and inter-arrival times (duration of vacancies) were simulated according to these (empirically measured) statistics for all sensor nodes. In this way, we generate on-the-fly synthetic traces $s_{i}\left(t\right)$ for all sensor nodes, which we deem reasonably well representative of real parking patterns. In Figure 2a,b, we respectively show the mean occupancy rate as a function of the hour of the day measured from the Worldsensing dataset and from the synthetic parking traces we obtained through the simulator. The occupancy rate for a certain hour of the day is defined as $T_{\mathrm{ON}}^{i}/\left(T_{\mathrm{ON}}^{i}+T_{\mathrm{OFF}}^{i}\right)$ and is averaged over all sensors i=1,⋯,N by only considering the parking states within that hour. In these plots, the occupancy metric is shown for the real data traces and for the synthetic ones. The occupancy curves in these two plots are reasonably close and suggest that our event-based simulator provides a fairly accurate approximation of mean occupancy figures across the entire day (both for weekends and weekdays). We further observe that higher accuracies in the simulated model can be achieved by decreasing the time granularity, e.g., by tracking the relevant statistics for each minute of the day. This however has two main drawbacks: the first is that the computational complexity substantially increases, and the second is that longer observation intervals would be required for an accurate estimation of the pdfs. Overall, an hour granularity provided a good tradeoff between complexity and accuracy.

This simulation tool is instrumental to selectively set an unusual behavior during certain days (e.g., weekends, holidays), no-activity or unusually high activity for certain nodes, time periods or geographical areas (e.g., to mimic street maintenance or node failures). This entails the addition of the number, location and occurrence time of outlier nodes in a controlled way and the assignment of different statistics to several clusters of (possibly non-adjacent) nodes. This allows for the fine tuning and the precise assessment of the considered clustering algorithms in terms of outlier detection and classification performance, as we show in Section 5.

## 4. A Simple Anomaly Detection Approach

Anomaly detection (also referred to as outlier detection) refers to the identification of data points, events or observations that do not conform to expected patterns.

As a first naive approach to the detection of outliers, we may use the cumulative distribution functions (cdf) of r.v.’s $t_{\mathrm{ON}}$ and $t_{\mathrm{OFF}}$, which are respectively defined as $F_{\mathrm{ON}}\left(\tau \right)=\mathrm{Prob}\left\{t_{\mathrm{ON}}\leq \tau \right\}$ and $F_{\mathrm{OFF}}\left(\tau \right)=\mathrm{Prob}\left\{t_{\mathrm{OFF}}\leq \tau \right\}$. In particular, we consider the tail of such distributions that, e.g., for $t_{\mathrm{ON}}$ is defined as $P\left(t_{\mathrm{ON}}>\tau \right)=1-F_{\mathrm{OFF}}\left(\tau \right)$, where *τ* is the duration of a parking event. Hence, we assess whether a parking event is an outlier through the following rules:

(1) a threshold ξ∈(0,1) is set and,
(2) a parking event with duration *τ* is flagged as anomalous if $P(t_{\mathrm{ON}}>\tau )<\xi$.

For each parking sensor, tail distributions are empirically evaluated for each time slot (considering an hourly granularity) and for each day of the week. They are subsequently used to flag outliers as we just explained. Note that this algorithm can be either implemented in a centralized fashion, i.e., performing the required processing in the backend servers (upon the collection of all data) or in a distributed manner, i.e., each sensor autonomously estimates the tail distributions for the monitored parking space and uses them to flag its own outliers.

Before delving into the discussion of the results, two definitions are in order.

**Definition** **1.** Accuracy: We define accuracy as the ratio between the number of correctly-detected outliers over the total number of detected outliers.

**Definition** **2.** Detection rate: We define the detection rate as the ratio between the number of correctly-detected outliers over the total number of outliers in the dataset.

We would like to reach high accuracies, meaning that most of the outliers detected by our algorithm indeed represent real deviations from the expected behavior and, as such, may flag situations requiring our attention, such as broken sensor units, high traffic conditions, street maintenance, fairs, and so forth. However, we would also like to obtain high detection rates, meaning that all of the outliers are ideally detected.

**Figure 3:**
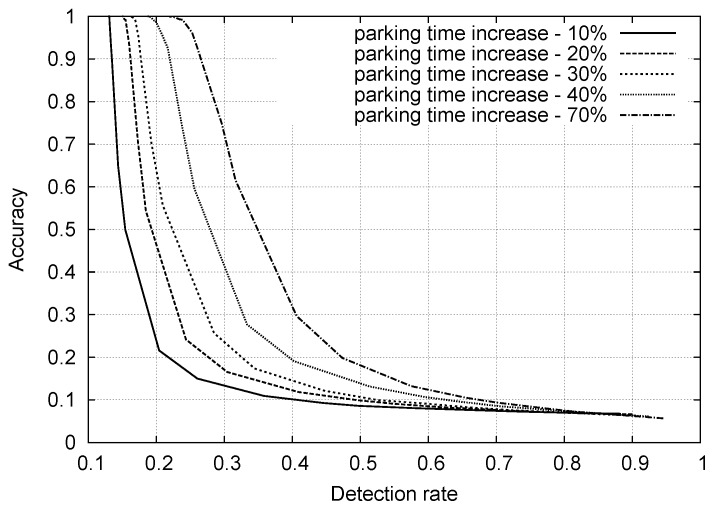
Anomaly detection: accuracy vs. detection rate: month of January 2015. Different curves correspond to specific increases in the parking times, from 10% to 70% for 20% of the parking nodes.

In Figure 3, we plot the accuracy vs. the detection rate for the above naive scheme, where we varied threshold *ξ* as a free parameter in (0,1). This plot was obtained by considering sensor traces from the entire month of January 2015, where we modified the statistical behavior of 74 parking sensors (20% of the total population), increasing the parking duration of all of their events by a given percentage, as indicated in the plot (one curve for each percentage, from 10% to 70%). For ξ→0, all curves move toward the upper-left corner of the plot, providing high accuracy, but the detection rate correspondingly decreases. On the other hand, as ξ→1, we reach high detection rates, but the accuracy becomes unacceptably low. In general, this first approach has the main advantages of being simple, computationally inexpensive (once the empirical distributions are computed) and amenable to a distributed implementation. It however has the major drawback that a satisfactory tradeoff between the accuracy and detection rate is hard to achieve. Better classifiers are designed and evaluated in the following sections.

## 5. Advanced Classification Techniques

In this section, we describe some advanced classification techniques that will lead to an improved performance with respect to the approach of Section 4. To this end, we define the concept of outliers (see Section 5.1) and introduce a suitable set of features that are utilized by the subsequent algorithms for an effective classification of the parking sensors (see Section 5.2). After that, we describe standard classification approaches, namely *k*-means, EM and DBSCAN (see Section 5.3), and a new clustering algorithm based on self-organizing maps (see Section 5.4).

### 5.1. Discussion on Outliers

Outliers are generally defined as data points that globally have the least degree of similarity to the dataset they belong to, and for our classification task, a data point corresponds to the time series generated by a certain sensor, which represents the behavior of the associated parking space. With the previous naive technique, we overlooked the fact that the time series in the considered parking data span a wide range of behaviors, which makes it difficult to identify outliers by looking at the dataset as a whole. Nevertheless, we recognized that there exist time series with similar characteristics, which allows splitting the data into clusters. We then found that within each cluster, parking sequences are much more homogeneous, and in turn, outliers are easier to identify. For this reason, in the remainder, instead of looking for outliers by looking at the complete dataset at once, we first split it into clusters and then perform outlier identification inside each of them.

In addition, we note that assessing whether a certain sensor is an outlier strongly depends on the definition one uses for similarity. If, for example, we were to compare parking sequences only based on the average duration of their parking events, then two sequences with the same average event duration and different event frequency would be treated as similar. Of course, this is acceptable as long as our application does not need to track event frequency. For instance, event duration is all that matters to assess whether the parking time has expired, in which case, a fine is to be issued to the car owner.

Hence, the definition of the features of interest is crucial for the correct ascertainment of outliers, and we also need to define a similarity metric over these features. In the following sections, we identify a suitable feature set for our classification problem and quantify the concept of similarity between feature vectors.

### 5.2. Features for Parking Analysis

In machine learning and pattern recognition [27], a feature can be defined as an individual measurable property of a phenomenon being observed. Informative, discriminating and independent features are key to the design of effective classification algorithms. For their definition, we consider, for each parking sensor *i*, the following statistical measures:

(1) Sensor occupation (SO): accounts for the amount of time during which $s_{i}\left(t\right)=1$, i.e., the corresponding parking space is occupied.
(2) Event frequency (EF): accounts for the number of parking events per unit time.
(3) Parking event duration (PD): measures the duration of parking events.
(4) Vacancy duration (VD): measures the duration of vacancies.

We computed the hourly average trend for each of the above measures, considering two classes, (cl1) weekdays and (cl2) weekends, and averaging the data points corresponding to the same hour for all of the days in the same class. For each statistical measure, this leads to 24 average values, where the value associated with hour t∈{1,⋯,24} for Classes cl1/cl2 is obtained averaging the measure for hour *t* across all days of the same class. Thus, hourly sensor occupation functions were obtained for each hour of the day t∈{1,⋯,24} for Classes cl1 and cl2, which we respectively denote by $m_{\mathrm{SO}}^{1}\left(t\right)$ and $m_{\mathrm{SO}}^{2}\left(t\right)$. Similar functions were obtained for the remaining measures, i.e., $m_{\mathrm{EF}}^{*}\left(t\right)$, $m_{\mathrm{PD}}^{*}\left(t\right)$, $m_{\mathrm{VD}}^{*}\left(t\right)$, where ${}^{*}$ is either one (cl1) or two (cl2). We remark that all functions $m_{a}^{b}\left(t\right)$ have been normalized through $m_{a}^{b}\left(t\right)\leftarrow \left(m_{a}^{b}\left(t\right)-m_{\min}\right)/\left(m_{\max}-m_{\min}\right)$, where $m_{\max}$ and $m_{\min}$ respectively represent the minimum and maximum elements in the dataset for measure a∈{SO,EF,PD,VD} and class b∈{1,2}. Other normalizations were also evaluated, but this one led to the best classification results.

This leads to a total of 2×24=48 average values for each measure (two classes and 24 h per class), which amounts to a total of 4×48=192 values to represent the four statistical measures that we utilize to characterize the parking behavior of a sensor. Note that we purposely decided to separately process data from weekdays and weekends. This is because parking data from these two classes exhibits a significantly different behavior, and explicitly accounting for this fact increased the precision of our algorithms (although at the cost of a higher number of feature elements).

**Figure 4:**
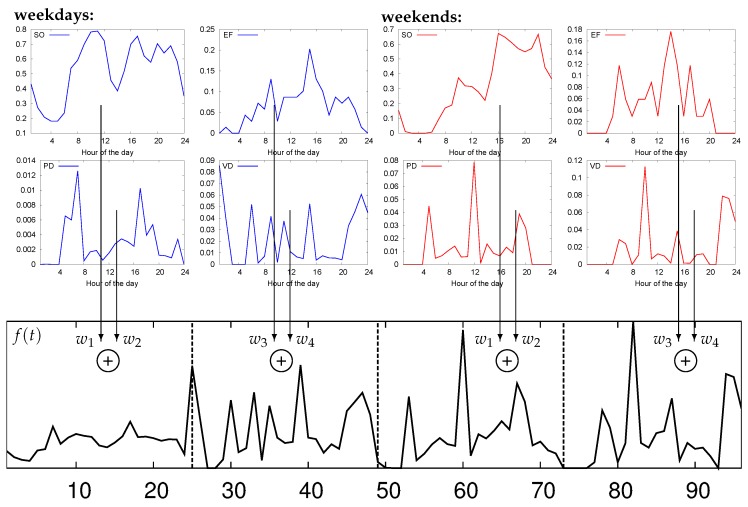
Feature function f(t): calculation example for a typical parking sensor. The feature vector $x_{i}={\left[x_{i1},\cdots ,x_{iK}\right]}^{T}$ for sensor *i* is obtained as $x_{it}\leftarrow f\left(t\right)$ for t=1,⋯,K.

Thus, the eight functions (four per class) are computed for each sensor in the parking deployment, and their weighted sum is computed using suitable weights $w_{1},w_{2},w_{3},w_{4}$ with $\sum_{k=1}^{4}w_{k}=1$ and $w_{k}\in \left[0,1\right]$. In this way, we obtain a single feature function f(t) (see Equation (2)) consisting of 96 average values: the first 48 values are representative of the average hourly measures from Class cl1, whereas the second 48 values represent the hourly measures from Class cl2. The weights determine the relative importance of each statistical measure; their correct assignment is crucial to obtain a good classification performance, as we show in Section 6.2. The feature function f(t) is obtained as:

(2) $$f\left(t\right)=\left\{\begin{array}{cc} w_{1}m_{\mathrm{SO}}^{1}\left(t\right)+w_{2}m_{\mathrm{PD}}^{1}\left(t\right) & t\in \{1,\cdots ,24\}\\ w_{3}m_{\mathrm{EF}}^{1}\left(t^{\prime }\right)+w_{4}m_{\mathrm{VD}}^{1}\left(t^{\prime }\right) & t\in \{25,\cdots ,48\}\\ w_{1}m_{\mathrm{SO}}^{2}\left(t^{\prime }\right)+w_{2}m_{\mathrm{PD}}^{2}\left(t^{\prime }\right) & t\in \{49,\cdots ,72\}\\ w_{3}m_{\mathrm{EF}}^{2}\left(t^{\prime }\right)+w_{4}m_{\mathrm{VD}}^{2}\left(t^{\prime }\right) & t\in \{73,\cdots ,96\} \end{array}\right.$$

where $t^{\prime }=\left((t-1)\;\mathrm{mod}\;24\right)+1$. We remark that f(t) is sensor-specific, i.e., one such function is computed for each parking sensor. Furthermore, for each parking sensor *i*, this function defines a feature vector $x_{i}={\left[x_{i1},x_{i2},\cdots ,x_{iK}\right]}^{T}\in R^{K}$ of K=96 elements that are used to train the clustering algorithms of the next sections. Specifically, for sensor *i*, we set $x_{it}\leftarrow f\left(t\right)$, t=1,⋯,K, where $m_{a}^{b}\left(t\right)$ in f(t) is computed from measures of that sensor. A feature function example from our parking dataset is shown in Figure 4.

### 5.3. Selected Clustering Techniques from the Literature

Many clustering algorithms were proposed over recent decades; see [28] for a literature survey. From this paper, an operational definition of clustering is: “given a representation of *n* objects, find *k* groups based on a measure of similarity such that the similarities between objects in the same group are high, while those between objects in different groups are low”. Note that our problem is in general more complex, as the number of clusters (data classes) is not known beforehand, but has to be inferred from the data. In this section, we review three clustering techniques from the literature, i.e., *k*-means, EM and DBSCAN. These tackle the clustering problem from quite different angles and will be considered for the performance evaluation of Section 6. As will be shown, while they all detect similar clusters, none of them is entirely satisfactory, as outliers go most of the times undetected. Our SOM-based approach solves this.

*k*-means: *k*-means is a very popular clustering technique [4], which is successfully used in many applications. Basically, given *n* input data vectors $x_{i}={\left[x_{i1},x_{i2},\cdots ,x_{id}\right]}^{T}$, where $x_{i}\in R^{d}$ and i=1,⋯,n, the aim is to determine a set of k≤n vectors, referred to as cluster centers, that minimizes the mean squared distance from each vector in the set to its nearest center. A popular heuristic to achieve this is Lloyd’s algorithm [29]. First, *k*-means is initialized by choosing *k* cluster centers, called centroids. Hence, it proceeds by alternating the following steps until convergence:

(1) Compute the distances between each input data vector and each cluster centroid.
(2) Assign each input vector to the cluster associated with the closest centroid.
(3) Compute the new average of the points (data vectors) in each cluster to obtain the new cluster centroids.

The algorithm stabilizes when assignments no longer change. The clustering results provided by this technique depend on the quality of the starting points of the algorithm, i.e., the initial centroids. In this work, we consider the *k*-means++algorithm [30], which augments *k*-means with a low complexity and randomized seeding technique. This is found to be very effective in avoiding the poor clusters that are sometimes found by the standard *k*-means algorithm. Note also that *k*-means is here considered due to its popularity as a baseline clustering scheme. Nevertheless, we stress that the number of clusters *k* has to be known beforehand, which is not the case with real (unlabeled) parking data. Hence, techniques that discover a suitable number of clusters in an unsupervised manner are preferable, and the ones that we treat in the following do so.

EM: EM is an unsupervised classification technique, which fits a finite Gaussian Mixture Model (GMM) on the provided input vectors $x_{i}$, i=1,⋯,N (one vector for each parking sensor), using the EM algorithm [6] to iteratively estimate the model parameters. Within the considered GMM, the number of mixtures equals the (pre-assigned) number of clusters, and each probability distribution models one cluster. A naive implementation of the algorithm requires to know beforehand the number of classes into which the dataset should be subdivided. Here, we implemented the procedure proposed in [6] through which the number of clusters is automatically assessed.

The steps involved in the considered EM clustering algorithm are:

(1) Initial values of the normal distribution model (mean and standard deviation) are arbitrarily assigned.
(2) Mean and standard deviations are iteratively refined through the expectation and maximization steps of the EM algorithm. The algorithm terminates when the distribution parameters converge or a maximum number of iterations is reached.
(3) Data vectors are assigned to the cluster with the maximum membership probability.

For the automatic assessment of the number of clusters, we proceeded by cross-validation. This starts by setting the number of clusters to one. Thus, the training set is split into a given number of folds (10 for the results in this paper). EM is then performed ten times with the ten folds. The obtained log likelihood values are averaged. If the log likelihood is increased when the number of clusters is increased by one, then the procedure is repeated. The Weka data mining software was used to this end [31].

DBSCAN: DBSCAN can be considered as the de facto standard unsupervised clustering technique [5]. A set of *n* points (real vectors in $R^{d}$) is taken as the input, and the objective is to group them into a number of regions. Differently from *k*-means, the number of clusters (regions) does not need to be specified a priori. Two parameters have to be set up prior to applying the algorithm, namely:

(1) *ε*: used to define the *ε*-neighborhood of any input vector x, which corresponds to the region of space whose distance from x is smaller than or equal to *ε*.
(2) MinPts: representing the minimum number of points needed to form a so-called dense region.

An input vector x is a core point if at least minPts points (including x) are within distance *ε* from it. The following applies: all of the points in the *ε*-neighborhood of a certain vector x are reachable from it, whereas no points are directly reachable from a non-core point. A point y is reachable from x if we can locate a path of points with endpoints y and x, and all subsequent points in the path are mutually reachable. All points that are not reachable from any other point are tagged as outliers. DBSCAN starts from an arbitrary starting point (vector). If its *ε*-neighborhood has a sufficient number of points, a cluster is initiated; otherwise, the point is considered as noise. Note that this rejection policy makes the algorithm robust to outliers, but has to be tuned through a careful adjustment of the two parameters from above, whose best setting depends on the input data distribution. If a point is found to be a dense part of the cluster, its *ε*-neighborhood is also part of the cluster. From the first point, the cluster construction process continues by visiting all of the nodes that are mutually reachable, until the density-connected cluster is completely found. After this, a new point is processed, reiterating the procedure, which can lead to the discovery of a new cluster or noise. The main advantages of the algorithm are that the number of clusters does not have to be known, that it can discover arbitrarily-shaped regions, that is nearly insensitive to the ordering of the input points and that, if properly tuned, it is robust to outliers. Drawbacks are related to the choice of the distance measure and to the choice of the two parameters *ε* and MinPts, especially when clusters have large differences in densities, as a single parameter pair is unlikely to be good for all clusters.

### 5.4. Classification Based on Self-Organizing Maps

Next, we present an original clustering algorithm that exploits the unsupervised learning capabilities of self-organizing maps to automatically discover clusters and to concurrently identify outliers. This algorithm is here proposed, fine-tuned and tested against synthetic datasets and real parking signals (see Section 6).

The SOM [8,9] is a neuro-computational algorithm that maps high-dimensional data into a one- or two-dimensional space through a nonlinear, competitive and unsupervised learning process. The SOM differs from other artificial neural networks as it uses a neighborhood function to preserve the topological properties of the input space [32]. It is trained through input examples, and the input space is mapped into a two-dimensional lattice of neurons, preserving the property that similar input patterns are mapped by nearby neurons in the map.

In this work, we consider one- and two-dimensional maps, which are respectively made of sequences of M×1 neurons and a lattice of ℓ=M×M neurons, with M>1. These maps become selectively tuned to the input patterns through an unsupervised (also referred to as competitive) learning process. As learning progresses, the neuron weights tend to become ordered with respect to each other in such a way that a significant coordinate system for different input features is created over the lattice. In other words, a SOM creates a topographic map of the input data space, where the spatial locations or coordinates of the neurons in the lattice correspond to a particular domain or intrinsic statistical feature of the input data. Remarkably, this is achieved without requiring any prior knowledge on the input distribution.

**Figure 5:**
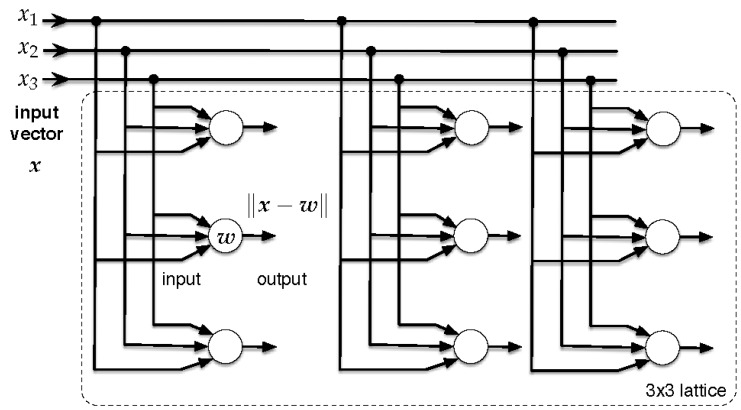
Flow diagram of the SOM training process.

With $X\subset R^{K}$, we indicate the feature (input) set, and we let $x_{i}\in X$ be the input feature vector associated with parking sensor i=1,⋯,N, where $x_{i}={\left[x_{i1},x_{i2},\cdots ,x_{iK}\right]}^{T}$; see Section 5.2. With *N*, we mean the number of sensors, and |X|=N. Let L be the lattice. Each neuron is connected to each component of the input vector, as shown in Figure 5. The links between the input vector and the neurons are weighted, such that the *j*-th neuron is associated with a synaptic-weight vector $w_{j}\in R^{K}$, where $w_{j}={\left[w_{i1},w_{i2},\cdots ,w_{iK}\right]}^{T}$.

**Figure 6:**
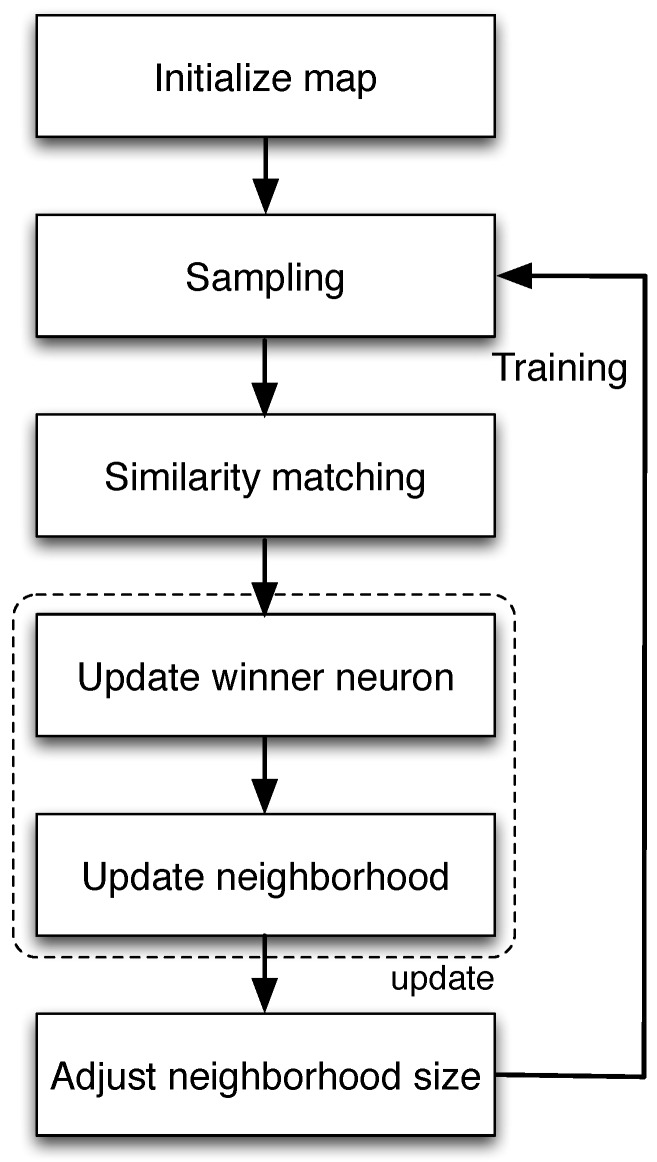
Flow diagram of the SOM training process.

**SOM-based clustering:** The clustering algorithm is summarized in Algorithm 1 and discussed in what follows, by identifying its main steps, i.e., training and clustering. Step 1: Training. The learning process occurs by means of showing input patters $x_{i}\in X$ to the SOM. Each time a new pattern is inputted to the map, the neurons compete among themselves to be activated, with the result that only one winning neuron is elected at any one time. To determine the winning neuron, the input vector $x_{i}$ is compared with the synaptic-weight vectors $w_{j}$ of all neurons. Only the neuron whose synaptic-weight vector most closely matches the current input vector according to a given distance measure (that we choose equal to the Euclidean distance, which is commonly used) dominates. Consequently, the weights of the winning neuron and of the nodes in the lattice within its neighborhood are adjusted to more closely resemble the input vector. The algorithm steps are (see also Figure 6) as follows.

**Algorithm 1** (SOM):Initialization: set the initial time step to n=0 and choose small random values for the initial synaptic-weight vectors $w_{j}\left(0\right)$, j∈L.Sampling: set n←n+1 and sample the training input pattern $x_{n}$, i.e., the feature vector from the *n*-th parking sensor.Similarity matching: find the winning neuron, whose index is $j^{☆}\left(n\right)$ at time step *n* by using the minimum-distance criterion:
(3)$$j^{☆}\left(n\right)=\arg\;\min_{j}∥x_{n}-w_{j}\left(n\right)∥,j\in L$$
where with ∥a−b∥, we mean the Euclidean distance between vectors a and b.Update: adjust the synaptic-weight vectors of all neurons by using the update equation:
(4)$$w_{j}\left(n+1\right)=w_{j}\left(n\right)+\eta \left(n\right)h_{ji}\left(n\right)\left(x_{n}-w_{j}\left(n\right)\right),$$
with j∈L, where η(n) is the learning rate parameter at iteration *n* and $h_{ji}\left(n\right)$ is the neighborhood function centered on $j^{☆}\left(n\right)$ at iteration *n*.Adjust neighborhood: adjust the neighborhood size ($h_{ji}\left(n\right)$), the learning rate (η(n)) and continue from Step 2 if $n<n_{\mathrm{iter}}$; stop otherwise.

A good and common choice for $h_{ji}\left(n\right)$ is the Gaussian function, whose span at time n=0 is chosen to cover all of the neurons in the lattice and is then reduced as the map is being trained. The reason for this is that a wide initial topological neighborhood, i.e., a coarse spatial resolution in the learning process, first induces a rough global order in the synaptic-weight vector values. Hence, during training, narrowing improves the spatial resolution of the map without destroying the acquired global order. The learning rate is also reduced as times goes by, and a sufficiently high number of iterations $n_{\mathrm{iter}}$ has to be chosen, so that all synaptic weights stabilize. Moreover, if the number of available input items is smaller than $n_{\mathrm{iter}}$, new examples can be resampled from the input data until convergence. See Chapter 9 of [32] for additional details.

Step 2: Clustering. Once the training is complete, the map has adapted to efficiently and compactly represent the feature space X. Clustering immediately follows using the trained map, as we now explain. The feature vectors $x_{i}\in X$ with i=1,⋯,N are fed as an input to the map. For each feature vector $x_{i}$, we use Equation (3) (with the final synaptic weights) to assess the winning neuron (also referred to as the activated neuron) in the map for this vector. With $j^{☆}$, we indicate the index of the winning neuron; see Equation (3). Clusters are constructed by grouping together the feature vectors returning the same index. It is then immediate to realize that the maximum number of clusters returned by SOM equals the number of neurons in the map, $M^{2}$, i.e., one cluster per neuron. We underline that in some cases, a small number of neurons may never be activated, i.e., be selected as the best fitting neuron for a certain input patter (vector). As a consequence, the number of clusters may be strictly smaller than $M^{2}$ (in our experimental validation, this number was never smaller than $M^{2}-2$). With this approach, the number of clusters is somewhat fixed in advance, as it strictly depends on the number of neurons in the map. Next, we propose an original algorithm that exploits SOM to self-assess the number of clusters according to a tree-splitting approach.

**Unsupervised SOM-based clustering**: The above SOM clustering approach requires knowing in advance the number of clusters in the dataset (i.e., the number of neurons in the SOM map). Next, we devise an unsupervised clustering algorithm that does not need to know in advance the number of data classes. We do this by taking inspiration from [33,34,35]: as done in these papers, instead of clustering data through an agglomerative approach, we adopt a divisive approach, i.e., we start from a big cluster containing the entire dataset, and we iteratively partition this initial cluster into progressively smaller ones. A SOM map with only two neurons is utilized as a non-linear classifier to split clusters into two subsets. SOM has a higher discriminant power than the linear-discriminant functions of [33]. Furthermore, we use a local-global adaptive clustering procedure, similar to that in [36], whose cornerstone is the self-similarity found in the global and local characteristics of many real-world datasets. Specifically, we assess a correlation measure among the features of the entire dataset (global metric) and those of the smaller clusters obtained at a certain step of the algorithm (local metrics). Hence, global and local metrics are compared to determine when the current clusters have to be further split. Prior to describing the algorithm, we introduce the following concepts:

- Data point: The input dataset is composed of *N* data points, where “data point” *i* is the feature column vector $x_{i}\in X$ associated with the parking sensor i=1,⋯,N. These vectors are conveniently represented through the full feature matrix $X=[x_{1},\cdots ,x_{N}]$. With $X_{p}$, we mean a submatrix of X obtained by collecting *p* columns (feature vectors), not necessarily the first *p*. A generic cluster C containing *p* elements is then uniquely identified by a collection of *p* sensors and by the corresponding feature matrix $X_{p}$.
- Cluster cohesiveness: Consider a cluster C with *p* elements, and let $X_{p}$ be the corresponding feature matrix. We use a scatter function as a measure of its cohesiveness, i.e., to gauge the distance among the cluster elements and its mean (centroid). The centroid of $X_{p}=\left[x_{1},\cdots ,x_{p}\right]$ is computed as: $\mu_{p}=\left(\sum_{j=1}^{p}x_{j}\right)/p$. The dispersion of the cluster members around $\mu_{p}$ is assessed through the sample standard deviation:
  (5) $$\sigma \left(X_{p}\right)=\sqrt{\frac{1}{p-1}\sum_{j=1}^{p}{∥x_{j}-\mu_{p}∥}^{2}}\;,$$
  where ∥x∥ is the Euclidean norm of vector x. Likewise, the dispersion of the full feature matrix X is denoted by σ(X), and we define a further threshold $\sigma_{\mathrm{th}}=\gamma \sigma \left(X\right)$ for a suitable γ∈[0,1].
- Global vs. local clustering metrics: In our tests, we experimented with different metrics, and the best results were obtained by tracking the correlation among features, as we now detail. We proceed by computing two statistical measures: (1) a first metric, referred to as global, is obtained for the entire feature matrix X; (2) a local metric is computed for the smaller clusters (matrix $X_{p}$).
  (1) Global metric: Let X be the full feature matrix. From X, we obtain the N×N correlation matrix $C=\{c_{ij}\}$, where $c_{ij}=\mathrm{corr}\left(x_{i},x_{j}\right)$. Thus, we average C by row, obtaining the *N*-sized vector $\bar{c}={\left[{\bar{c}}_{1},\cdots ,{\bar{c}}_{N}\right]}^{T}$, with ${\bar{c}}_{i}=\left(\sum_{j=1}^{N}c_{ij}\right)/N$. We respectively define $\mathrm{stdev}(\bar{c})$ and $\mathrm{mean}(\bar{c})$ as the sample standard deviation and the mean of $\bar{c}$. We finally compute two global measures for matrix X as:
    (6) $$\begin{array}{ccc} \mathrm{meas}1(X) & = & \mathrm{stdev}(\bar{c})\\ \mathrm{meas}2(X) & = & \mathrm{mean}(\bar{c})\;. \end{array}$$
  (2) Local metric: The local metric is computed on a subsection of the entire dataset, namely on the clusters that are obtained at runtime. Now, let us focus on one such cluster, say cluster C with |C|=p. Hence, we build the corresponding feature matrix $X_{p}$ by selecting the *p* columns of X associated with the elements in C. Thus, we compute the correlation matrix of $X_{p}$, which we call $C_{p}$, and the *p*-sized vector ${\bar{c}}^{\prime }={\left[{\bar{c}}_{1}^{\prime },\cdots ,{\bar{c}}_{p}^{\prime }\right]}^{T}$, obtained averaging $C_{p}$ by row as above. The local measures associated with matrix $X_{p}$ are:
    (7) $$\begin{array}{ccc} \mathrm{meas}1(X_{p}) & = & \mathrm{stdev}({\bar{c}}^{\prime })\\ \mathrm{meas}2(X_{p}) & = & \min({\bar{c}}^{\prime })\;, \end{array}$$
    where $\min({\bar{c}}^{\prime })$ returns the smallest element in ${\bar{c}}^{\prime }$.
- Global vs. local dominance: We now elaborate on the comparison of global and local metrics. Let X and $X_{p}$ respectively be the full feature matrix and that of a cluster obtained at runtime by our algorithm. Global and local metrics are respectively computed using Equations (6) and (7) and are compared in a Pareto [37] sense as follows. We say that the global metric (matrix X) dominates the local one (matrix $X_{p}$) if the following inequalities are jointly verified:
  (8) $$\begin{array}{ccc} \mathrm{dominance} & & \\ \mathrm{meas}1(X) & > & \mathrm{meas}1(X_{p})\\ \mathrm{meas}2(X) & < & \mathrm{meas}2(X_{p})\;. \end{array}$$
  For the first measure (meas1), global dominance occurs when the global standard deviation is strictly larger than the local one. For the second measure (meas2), global dominance occurs when the global mean is smaller than the local minimum. Full dominance occurs when the two conditions are jointly verified. Note that when the local metrics are non-dominated by the global ones, this means that the vectors in the associated cluster either have a larger variance or that the minimum correlation in the cluster is larger than the average correlation in the entire dataset. In both cases, the local measures are not desirable, as the cluster still has worse correlation properties than the full data. Hence, we infer that the cluster has to be split, as it either contains uncorrelated data points or at least one outlier.

Our unsupervised SOM-based clustering technique is detailed next.

**Algorithm 2** Unsupervised SOM clustering:
Initialization: Initialize an empty cluster set A=∅; assign all of the parking sensors i=1,⋯,N to a single cluster; mark it as non-finalized; and add it to set A. Initialize a second empty set B=∅, used to collect the clusters created by the algorithm through successive partitioning. Compute the global metrics meas1(X) and meas2(X).Evaluation: If set A is empty, go to termination. Otherwise, pick a cluster at random from A and compute its local metrics. Let C and $X_{p}$ respectively be this cluster and the associated feature matrix. If either of the following conditions is verified, (c1) the local metrics $\mathrm{meas}1(X_{p})$ and $\mathrm{meas}2(X_{p})$ are non-dominated by the global ones or (c2) the cohesiveness of C is such that $\sigma \left(X_{p}\right)>\sigma_{\mathrm{th}}$, then perform bipartition on cluster C (Step 2a below); otherwise, perform finalization (Step 2b below).(a)Bipartition: Remove C from A, and split it into two new clusters using a 2×1 SOM (involving Step 1, Training, and Step 2, Clustering). Mark the two clusters so obtained as non-finalized and add them to set A. Return to the evaluation step.(b)Finalization: Remove cluster C from A; mark it as finalized; and add it to B. This cluster will no longer be evaluated. Return to the evaluation step.Termination: When all clusters have been finalized, merge all single-element clusters in B in a single set, which is referred to as the outlier cluster. At this stage, B contains the final clusters.Polishing: A final polishing (or merging) procedure is executed on the final clusters in B. The aim of this is to join clusters that were separated to isolate outliers, but that actually contain points that are very close to one another. The polishing procedure is described below.

Polishing: The above cluster splitting procedure is very effective in isolating outliers, and these are usually moved onto clusters containing a single element (singletons). Nevertheless, some of the resulting non-singleton clusters may contain data points that are very close to one another (according to, e.g., the Euclidean distance metric), and this is because separating a single outlier from a cluster may at times require multiple splitting steps, which may entail scattering a uniform cluster into multiple, but (statistically) very similar ones. To solve this, we use a final polishing procedure to rejoin (merge) similar clusters. A similar strategy was originally proposed in [38]. The merge works as follows: Let $\left(C_{1},C_{2}\right)$ be a cluster pair in B. We first evaluate their union $C=C_{1}\cup C_{2}$, from which we obtain $X_{p}$, the feature matrix of C, and its cohesiveness $\sigma (X_{p})$. We compute the cohesiveness for each cluster pair in B, and we merge the two clusters with the smallest one. This is repeated until a certain stopping condition is met. Two stopping conditions can be considered: (s1) we keep merging until the number of cluster is equal to a preset number *k*; (s2) we keep merging until in B there are no cluster pairs $\left(C_{1},C_{2}\right)$ with $\sigma \left(X_{p}\right)<\sigma_{\mathrm{th}}$.

Discussion: Algorithm 2 iteratively splits the feature set X into smaller subsets, based on the relation between global and local metrics and on a local target cohesiveness, $\sigma_{\mathrm{th}}$. SOM classifiers are utilized due to their self-tuning and non-linear characteristics, and decision (Voronoi) regions to separate the dataset into clusters are obtained in a fully unsupervised manner. We observe that splitting the data points (feature vectors) into progressively smaller subsets allows for a progressively increasing precision in the classification, i.e., at the beginning, we perform a coarse classification that is subsequently refined in the following iterations. Most importantly, the decision as to whether to split is made based on self-similarity considerations, using local vs. global metrics, but also on the expected cohesiveness of the clusters. For real data, we found the self-similarity principle to be especially important (and often the only one that was used), but for more regular (synthetic) data patterns (e.g., same statistics across days and regularly spaced in terms of average measures), the second strategy had a higher impact. The best performance was obtained through a combination of these two approaches, and it has shown a robust behavior of the clustering algorithm in all of the considered settings. The final algorithm requires one to set the single parameter $\sigma_{\mathrm{th}}$ (*γ*), which represents our clue about the target cohesiveness of the true clusters. Note that the same $\sigma_{\mathrm{th}}$ is used in the splitting and in the polishing phases. The DBSCAN parameters *ε* and MinPts play a similar role.

## 6. Numerical Results

In this section, we fine tune and evaluate the previously-discussed clustering techniques. In Section 6.1 and Section 6.2, we use synthetic parking traces of increasing complexity, by adjusting the clustering parameters so as to obtain the best possible classification performance. Note that working with synthetic data is very valuable, as it provides a ground truth to assess the quality of the clusters identified by the schemes. Specifically, in Section 6.1, we test the classification performance for an increasing number of clusters, whereas in Section 6.2, we keep the number of clusters fixed, but we use synthetic signals exhibiting complex statistics, with parameters changing hourly across a day and differing between weekdays and weekends. These signals are designed in an attempt to mimic, as accurately as possible, those in the Worldsensing deployment. Hence, in Section 6.3, the selected clustering techniques are tested with real parking data.

Performance measures: The two most widely-adopted metrics to assess the goodness of a classifier are its precision *P* and recall *R*. For our multi-class problem, their calculation entails the computation of a so-called confusion matrix $Z=\{z_{ij}\}$, as follows [39]. In general, let *k* be the number of classes (clusters), and let $z_{ij}$ be the number of data points classified of class *i* that actually belong to class *j*, with i,j=1,⋯,k. Therefore, $z_{ii}$ are the points that are correctly classified (the true positives), whereas $z_{ij}$, with i≠j are misclassified points. The confusion matrix is Z; the precision associated with class *i* is computed as the fraction of events that were correctly classified of class *i* ($z_{ii}$) out of all instances where the clustering algorithm declared that a point belongs to class *i*, i.e., $P_{i}=z_{ii}/\sum_{j=1}^{k}z_{ij}$; the recall is instead the ratio between the number of points that were correctly classified of type *i* divided by the total number of type *i* events, i.e., $R_{i}=z_{ii}/\sum_{j=1}^{k}z_{ji}$. As is customary in the evaluation of classifiers, precision and recall are often combined into their harmonic mean [40], which is called the F-measure and is used as the single quality parameter. The F-measure associated with class *i* is thus:

(9) $$F_{i}=2P_{i}R_{i}/\left(P_{i}+R_{i}\right)\;.$$

The weighted F-measure (F) is finally obtained as a weighted average of the classes’ F-measures, weighted by the proportion of how many points are in each class. One last consideration is in order. We deal with an unsupervised classification problem, and in turn, although the use of synthetic traces allows controlling the real number of classes *k*, the clustering schemes may split the data points into $k^{\prime }\neq k$ sets. The F-measure calculation has to be modified to take this into account, and we did so by computing the weighted F-measure (through the above procedure) on the $k^{\prime \prime }=\min\left(k^{\prime },k\right)$ clusters identified by the algorithms that most closely matched the actual *k* ones (specifically, precision $P_{i}$ and recall $R_{i}$ are computed for each cluster $i=1,\cdots ,k^{\prime \prime }$, where these are the clusters that most closely match the actual *k* clusters in the dataset, obtaining $P_{i}=z_{ii}/\sum_{j=1}^{k^{\prime }}z_{ij}$ and $R_{i}=z_{ii}/\sum_{j=1}^{k^{\prime }}z_{ji}$, where $k^{\prime }$ is the number of clusters found by the algorithm).

### 6.1. Synthetic Data: Classification Performance with Varying Number of Clusters

For the results in this section, we artificially created a dataset with a predefined number of clusters, each of them featuring specific distributions for parking event durations (r.v. $t_{\mathrm{ON}}$) and vacancies (r.v. $t_{\mathrm{OFF}}$). The number of clusters is *k*, with k∈{2,3,⋯,20}, and the total number of parking sensors is kept fixed and equals N=370 (i.e., N/k sensors per cluster on average). The sensors in the first cluster have the lowest average parking time, i.e., $T_{ON}=10\;\min$, and the highest $T_{OFF}=600\;\min$. The last cluster contains sensors with the highest $T_{ON}$ and the lowest $T_{OFF}$. These values are inferred from the range of parking times and vacancies in the real dataset. The intermediate clusters have evenly-spaced $\left(T_{ON},T_{OFF}\right)$ pairs in the range (10, 600) min in a way that $T_{ON}$ increases from 10 to 600 min for an increasing *k*, whereas $T_{\mathrm{OFF}}$ correspondingly decreases from 600 to 10 min. The standard deviation is kept fixed at σ=30 min for all of the sensors and all values of *k*. For each *k*, the final multi-dimensional synthetic signal is obtained generating six months of parking events for each of the 370 sensors. While resembling real parking behaviors, this first dataset is much simpler than what we may expect in a real deployment. In fact, in real settings, parking statistics change on a hourly basis and differ from day to day. Although we consider more complex datasets in the following sections, we deem this evaluation meaningful, as it allows a preliminary assessment of the baseline performance of the selected clustering schemes.

The feature weights $w_{1},w_{2},w_{3},w_{4}$ are optimized for each clustering algorithm, so that it delivers its best possible classification performance for k=5 clusters. We did so because k=5 is the typical number of clusters that we have seen in real deployments, based on our inspection of real parking data. Hence, the weights are kept constant for all of the considered values of *k*, and the clustering parameters of each algorithm are optimized, by recalling that *k*-means does not require any parameters to be set, but takes the actual number of clusters *k* as input. Note that, as *k* increases, the correct classification of the dataset becomes increasingly difficult, posing serious challenges to all of the clustering techniques, as: (1) the number of sensors per cluster decreases, leading to fewer examples for each class and (2) the sensors belonging to neighboring clusters become more difficult to separate out, as the differences in their patterns become less pronounced.

**Figure 7:**
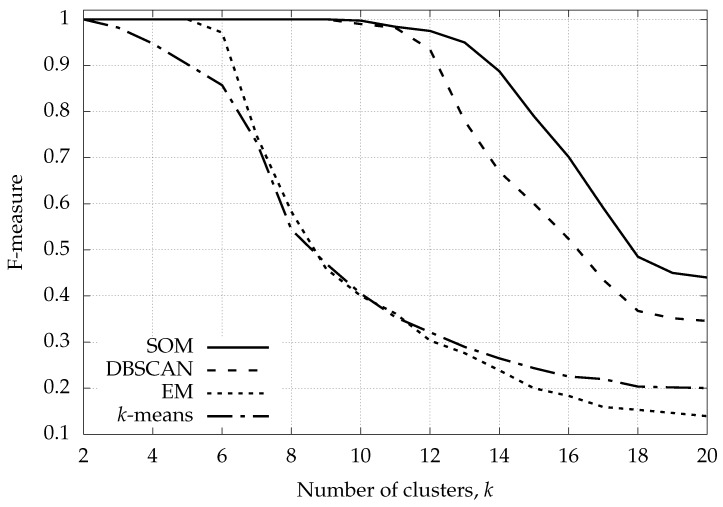
Weighted F-measure for the four selected clustering techniques vs. the number of clusters *k*.

The weighted F-measure for the clustering algorithms of Section 5 is shown in Figure 7. Each point in this plot was obtained by averaging over a number of experiments so that its 95% confidence interval falls within 1% of the (plotted) average F-measure. As shown in this figure, although *k*-means++ knows the exact number of clusters *k* in advance, it is not a good clustering solution. In fact, it fails to correctly classify the input data even when the number of clusters is low, i.e., smaller than five, and obtains a flawless classification only for k=2. EM does a better job, being able to perfectly classify the parking traces up to and including k=5, but it fails as *k* gets beyond this value, where its performance becomes comparable with that of *k*-means. DBSCAN performs much better, delivering perfect classifications up to and including k=9 and achieving a dramatic improvement over *k*-means and EM. This confirms the great ability of DBSCAN in classifying complex data, without knowing in advance the number of clusters (unsupervised clustering). What is shown in the plot is the best possible result that DBSCAN may deliver, as its parameters *ε* and MinPts were optimized for each *k*, so as to obtain the best classification performance. These parameters encode DBSCAN’s knowledge about the density and the variance of feature vectors inside the clusters. The solid curve in Figure 7 shows the weighted F-measure of our divisive SOM approach. The SOM-based algorithm shows superior performance, granting perfect classification up to and including k=10 and providing an F-measure improvement over DBSCAN ranging from 25% to 40% for k=13,⋯,20. SOM-based clustering has been optimized for each *k*, and this entails the adjustment of the sole parameter $\sigma_{\mathrm{th}}$.

### 6.2. Synthetic Data: Classification Performance with Outliers and Complex Statistics

**Figure 8:**
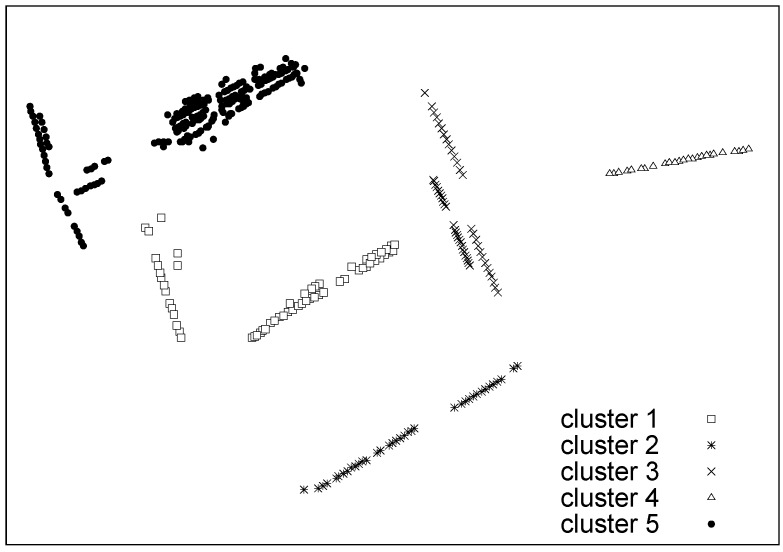
Visual representation of the five clusters considered in Section 6.2. The locations of the nodes correspond to those of the parking sensors in the considered Worldsensing deployment.

In this section, the clustering algorithms are fine-tuned (finding optimal weights and parameters) considering a second synthetic dataset that has been created to very closely resemble the statistics of real parking events. Hence, the optimized solutions are used with real data in the next Section 6.3. The N=370 sensor nodes in the deployment are split into five clusters, as visually represented in Figure 8, and all nodes within a cluster have the same statistical behavior. For each cluster, we have considered a different pair of Weibull pdfs. Specifically, the nodes in Cluster 1 were configured to produce an average parking duration as that dictated by the “Min” pdf in Table 2, whereas those of Cluster 5 reproduce the “Max” pdf. Clusters 2 to 4 were assigned three pdf pairs so as to obtain average parking durations evenly spaced between those of “Min” and “Max”. Once the $t_{\mathrm{ON}}$ pdf is assigned to a sensor, its $t_{\mathrm{OFF}}$ statistics is picked by matching the $T_{\mathrm{OFF}}$ that this sensor would show in the real parking dataset. In addition, parking statistics change on a hourly basis within the same day, and we differentiate between weekdays and weekends, so as to mimic as closely as possible the statistics of real data. Finally, 10% of the sensors generate parking events using statistical distributions that are typical of outliers. This is implemented to test the outlier detection capability of the algorithms.

**Figure 9:**
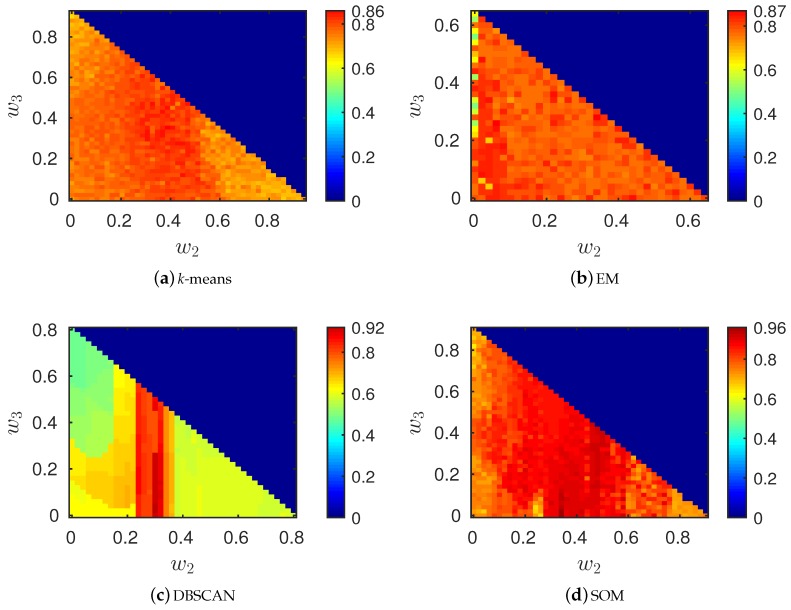
Optimal weights. In (**a**), the weights are $w_{1}=0.06$, $w_{2},w_{3}\in \left[0,0.94\right]$; in (**b**) the weights are $w_{1}=0.35$, $w_{2},w_{3}\in \left[0,0.65\right]$; in (**c**), the weights are $w_{1}=0.2$, $w_{2},w_{3}\in \left[0,0.8\right]$; in (**d**), the weights are $w_{1}=0.1$, $w_{2},w_{3}\in \left[0,0.9\right]$. In the heat maps, the weighted F-measure is represented with a color. The weights above the main diagonal are indicated with a dark blue color and are infeasible, as their sum is greater than one. Feasible weights lie below the main diagonal, and dark red means F-measure =1.

We simulated parking events for this setup running the *k*-means, DBSCAN, EM and SOM clustering algorithms for a number of instances, setting a different four-tuple $\left(w_{1},w_{2},w_{3},w_{4}\right)$ for each run, where $w_{4}=1-\left(w_{1}+w_{2}+w_{3}\right)$ and $w_{1},w_{2},w_{3}\in \left[0,1\right]$. At the end of each classification instance, we collected the synthetic parking traces from all nodes and checked whether the five clusters and the outlier sensors were successfully identified. We repeated this, spanning over the three weights $w_{1},w_{2}$ and $w_{3}$ and jointly searching for the best parameters (*ε* and MinPts for DBSCAN and *γ* for SOM). The final results of this search are shown in the heat maps of Figure 9, where we plot the F-measure as a function of $w_{2}$ and $w_{3}$, pre-assigning $w_{1}$ to the best weight for each scheme (we note that feasible weights are always below the main diagonal). The best classification results are obtained with the following parameters:

- *k*-means: $w_{1}=0.06$ and $w_{2}=w_{3}=0.3$.
- EM: $w_{1}=0.35$, $w_{2}=0.06$ and $w_{3}=0.26$.
- DBSCAN: $w_{1}=0.2$, $w_{2}=0.3$, $w_{3}=0.02$, ϵ=0.21 and MinPts =5.
- SOM: $w_{1}=0.1$, $w_{2}=0.34$, $w_{3}=0.04$ and γ=0.7 ($\gamma =\sigma_{\mathrm{th}}/\sigma \left(X\right)$).

From the results of Figure 9, we see that SOM provides better classification performance, and this is especially due to the fact that it more reliably identifies outliers. Furthermore, with respect to DBSCAN, we see that it generally provides good performance for a wider weight region. This fact is in general desirable for a clustering algorithm, as it amounts to an improved robustness against (unforeseen) changes in the statistics underpinning the data.

### 6.3. Classification Performance on Real Data

In this section, we apply the selected clustering techniques to the real parking data of Section 3.1. In Table 3, we show some occupancy statistics of the Worldsensing dataset over six months of data. Overall, the system is stable and well behaving: the hourly average occupancy remains mostly around 40%, and the system never reaches full capacity across all months. The maximum occupancy values are between 80% and 90%, with the highest peaks being during the winter holidays, as expected.

**Figure 10:**
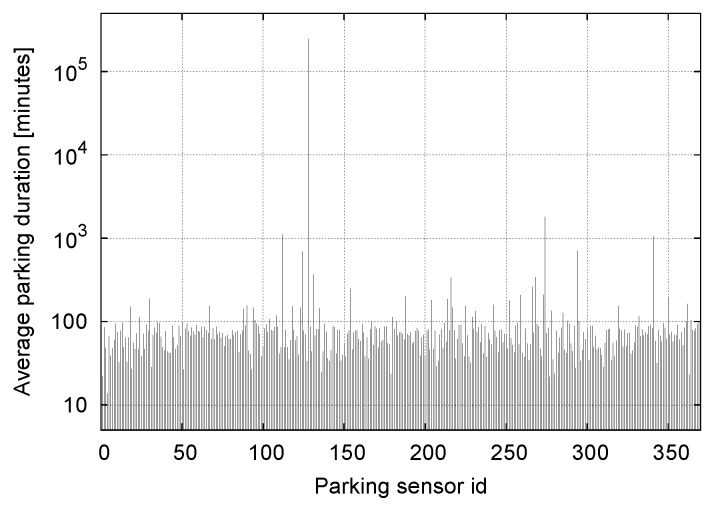
Average parking duration vs. parking sensor id.

However, some of the sensors do exhibit unexpected patterns, as can be observed from Figure 10, where we show the average duration of parking events as a function of the sensor identifier. From this plot, we see that at least four sensors reported very long parking events, i.e., on the order of days. A more careful inspection also revealed that, generally, these sensors reported only a few events across the entire observation period. At times, other sensors exhibited parking patterns that no Weibull model could fit. Parking sensors that presented either of these two characteristics, i.e., excessively long parking events or poor agreement with a Weibull pdf, were tagged as outliers.

**Figure 11:**
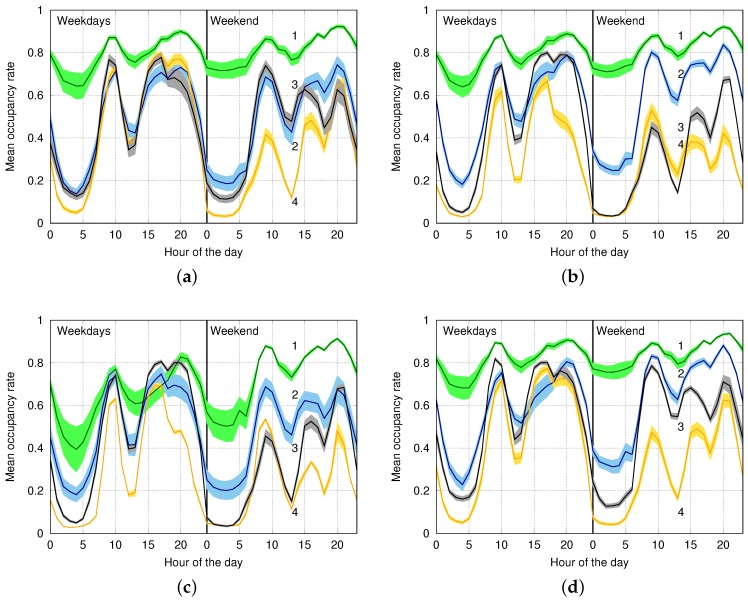
Clusters obtained on the Worldsensing dataset. (**a**) *k*-means; (**b**) EM; (**c**) DBSCAN; (**d**) SOM.

**Figure 12:**
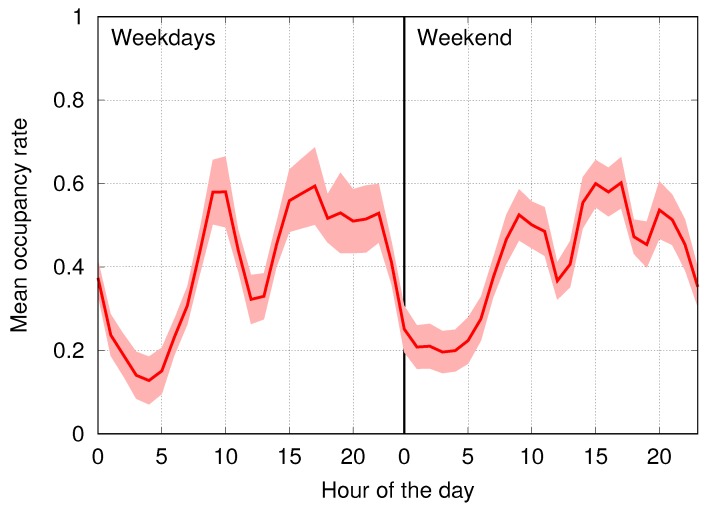
Outlier cluster found through SOM-based clustering.

In Figure 11, we plot the average occupancy curves for the four clusters generated on this dataset by *k*-means, EM, DBSCAN and SOM (Algorithm 2), which were configured with the weights and parameters found in Section 6.2. In these plots, the cluster identifiers have been indicated as a label on top of the corresponding curves, using the same numbering for the four schemes. We emphasize that SOM identifies an additional outlier cluster containing a total of 12 nodes, as shown in Figure 12, and that it successfully identifies all of the nodes that we manually tagged as outliers. On the other hand, we stress that *k*-means, DBSCAN and EM were unable to isolate these outliers, by instead spreading them over the four clusters. As a result, the first four clusters obtained by SOM (Figure 11d) have a smaller variance (represented through a shaded area around each curve) and are sharply separated out. This does not always occur for DBSCAN; see, e.g., Clusters 3 and 4 in Figure 11c. In addition, Clusters 1 and 2 in this plot show a higher variance with respect to their SOM counterparts, especially between 00:00 and 05:00, and Clusters 3 and 4 are almost overlapping within and around the same interval. For DBSCAN, the results of Cluster 1 look particularly impacted, as the corresponding occupancy rate is considerably lower than that of all of the other schemes, and this cluster is closer to the remaining ones. The results of *k*-means are clearly unsatisfactory, as Clusters 2 and 3 almost overlap. EM does a better job, delivering a good solution, with the only problem that Clusters 3 and 4 are now almost indistinguishable between 00:00 and 13:00 (weekend).

Similar considerations also hold for the remaining statistical parameters (EF, PD, VD) and also for the full feature vectors $x_{i}$, although differences in the feature space are more difficult to translate into practical considerations. Overall, the proposed SOM-based approach looks to be a promising technique, providing excellent classification results in all settings and also being quite effective in the identification of outlier nodes. A last observation is in order. In the present work, we did not explicitly measure the computational complexity of the algorithms as we target off-line learning strategies. In addition, with the considered dataset, the computation time is modest: we have measured computation times that never exceed one minute for the analysis of six months of data on a standard desktop computer with a 3.2-GHz quad-core Intel Core i5 processor with 8 GB RAM, using unoptimized MATLAB code. For considerably bigger datasets (big data), the presented algorithms have to be modified, for example using parallel computing techniques [41]. We leave these questions open for future research.

## 7. Conclusions

In this paper, we have investigated classification schemes for smart parking applications, focusing on the detection of outliers and on the joint and automated clustering of parking sensors as a function of their readings. Real data, from a commercial deployment, were used to understand the peculiarities of real-world parking events and then assess the effectiveness of selected classification approaches, namely *k*-means, expectation maximization clustering and DBSCAN. An original classification algorithm, based on self-organizing maps, was also proposed and proven to be superior to existing techniques, especially as concerns the detection of outliers. The present work is a first step toward data mining for smart parking applications, but several questions remain open to further explorations. We in fact believe that parking traces, besides being meaningful to street parking applications, also contain relevant information about how people behave, how neighborhoods are used (e.g., residential versus commercial) and may also reveal interesting facts about mobility, help implement traffic management solutions, etc. We leave these points open for future research.

## Figures and Tables

**Table 1:** Average, Max and Min values of the Weibull functions we fit on the dataset.

Parking	*Weekday (wd)*	*Weekend (we)*	*Mean (in Minutes)*
*Average*	λ=45.7422 κ=0.6039	λ=58.9885 κ=0.6313	$T_{\mathrm{ON}}\;=\;68.2438$ *(wd)* $T_{\mathrm{ON}}\;=\;83.3360$ *(we)*
*Max*	λ=124.8911 κ=0.8137	λ=121.0529 κ=0.8445	$T_{\mathrm{ON}}\;=\;139.8266$ *(wd)* $T_{\mathrm{ON}}\;=\;132.2398$ *(we)*
*Min*	λ=17.8723 κ=0.4245	λ=10.1799 κ=0.4119	$T_{\mathrm{ON}}\;=\;50.8284$ *(wd)* $T_{\mathrm{ON}}\;=\;31.2628$ *(we)*
**Vacancies**	***Weekday (wd)***	***Weekend (we)***	***Mean (in Minutes)***
*Average*	λ=112.4832 κ=0.8448	λ=101.3203 κ=0.7480	$T_{\mathrm{OFF}}\;=\;122.8511$ *(wd)* $T_{\mathrm{OFF}}\;=\;120.9045$ *(we)*
*Max*	λ=417.8844 κ=2.0947	λ=355.2186 κ=1.8366	$T_{\mathrm{OFF}}\;=\;370.1241$ *(wd)* $T_{\mathrm{OFF}}\;=\;315.6035$ *(we)*
*Min*	λ=15.2319 κ=0.4727	λ=9.5868 κ=0.4429	$T_{\mathrm{OFF}}\;=\;33.9791$ *(wd)* $T_{\mathrm{OFF}}\;=\;24.6376$ *(we)*

**Table 2:** Average Weibull parameters for the 5 synthetic clusters.

Weibull	*Weekday (wd)*	*Weekend (we)*	*Mean (in Minutes)*
*Cluster 1* *(Min)*	λ=2.8830 κ=4.9033	λ=4.7391 κ=3.8346	$T_{\mathrm{ON}}\;=\;2.6441$ *(wd)* $T_{\mathrm{ON}}\;=\;4.2853$ *(we)*
*Cluster 2*	λ=33.9250 κ=1.2681	λ=41.5004 κ=3.8024	$T_{\mathrm{ON}}\;=\;31.4959$ *(wd)* $T_{\mathrm{ON}}\;=\;37.5088$ *(we)*
*Cluster 3* *(Average)*	λ=45.7422 κ=0.6039	λ=58.9885 κ=0.6313	$T_{\mathrm{ON}}\;=\;68.2438$ *(wd)* $T_{\mathrm{ON}}\;=\;83.3360$ *(we)*
*Cluster 4*	λ=109.0669 κ=1.1866	λ=102.8083 κ=1.6052	$T_{\mathrm{ON}}\;=\;102.8975$ *(wd)* $T_{\mathrm{ON}}\;=\;92.1482$ *(we)*
*Cluster 5* *(Max)*	λ=390.601 κ=4.9137	λ=644.1756 κ=1.2876	$T_{\mathrm{ON}}\;=\;358.2768$ *(wd)* $T_{\mathrm{ON}}\;=\;596.1100$ *(we)*

**Table 3:** Average and Max occupancy per hour for the six months in the dataset (December 2014 to May 2015).

Occupancy Stat.	*December 2014*	*January 2015*	*February 2015*
*Avg/Hour*	44.49%	40.02%	42.04%
*Max/Hour*	91.22% 24 December 2014 at time 23:00	86.61% 23 January 2015 at time 20:00	87.22% 7 February 2015 at time 19:00
	***March 2015***	***April 2015***	***May 2015***
*Avg/Hour*	43.41%	40.04%	39.65%
*Max/Hour*	83.45% 21 March 2015 at time 19:00	88.60% 4 April 2015 at time 19:00	85.10% 9 May 2015 at time 19:00

## Abbreviations

DBSCAN: Density-Based Spatial Clustering of Applications with Noise
EF: Event Frequency
EM: Expectation Maximization
GMM: Gaussian Mixture Model
IoT: Internet of Things
PD: Parking Duration
PGI: Parking Guidance and Information
SO: Sensor Occupation
SOM: Self-Organizing Maps
SVDD: Support Vector Data Description
VD: Vacancy duration
WSN: Wireless Sensor Networks
